# A comprehensive review on biological activities of oxazole derivatives

**DOI:** 10.1186/s13065-019-0531-9

**Published:** 2019-02-04

**Authors:** Saloni Kakkar, Balasubramanian Narasimhan

**Affiliations:** Faculty of Pharmaceutical Sciences, Maharshi Dayanand University, Rohtak, 124001 India

**Keywords:** Oxazole derivatives, Antimicrobial, Anticancer, Antitubercular

## Abstract

The utility of oxazole as intermediates for the synthesis of new chemical entities in medicinal chemistry have been increased in the past few years. Oxazole is an important heterocyclic nucleus having a wide spectrum of biological activities which drew the attention of researchers round the globe to synthesize various oxazole derivatives and screen them for their various biological activities. The present review article aims to review the work reported on therapeutic potentials of oxazole scaffolds which are valuable for medical applications during new millennium.

## Background

**Fig. 1:**
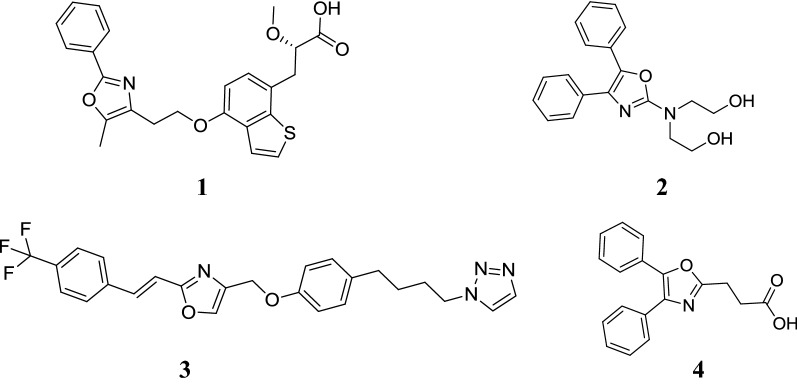
Marketed preparations containing oxazole

Heterocyclic systems are a part of large number of drugs and biologically relevant molecules. Often the presence of hetero atoms or groupings imparts preferential specificities in their biological responses. The chemistry and biological study of heterocyclic compounds has been interesting field for a long time [1] and oxazole is one such moiety which has gained attention in recent times due to its increasing importance in the field of medicinal chemistry. Oxazoles is a doubly unsaturated 5-membered ring having one oxygen atom at position 1 and a nitrogen at position 3 separated by a carbon in-between. It was first prepared in 1947, has a boiling point of 69 °C and is a stable liquid at room temperature [2]. Substitution pattern in oxazole derivatives play a pivotal role in delineating the biological activities like antimicrobial [3], anticancer [4], antitubercular [5] anti-inflammatory [6], antidiabetic [7], antiobesity [8] and antioxidant [9] etc. Oxazoles and its derivatives are a part of number of medicinal compounds (Fig. 1) which includes aleglitazar (**1**, antidiabetic), ditazole (**2**, platelets aggregation inhibitor), mubritinib (**3**, tyrosine kinase inhibitor), and oxaprozin (**4**, COX-2 inhibitor) [10].

From the literature, it was found that various types of review articles have been written on synthesized/natural oxazole compounds which are focused on their pharmacological significance in medicinal filed. Some of the reported review articles on oxazole moiety includes the work done by Joshi et al. who have presented a review on systematic scientific study of 1, 3-oxazole derivatives as a useful lead for pharmaceuticals [11], Swellmeen, prepared a review on 1,3-oxazole derivatives exhibiting their biological activities as antipathogenic [2] whereas Singh and Tilvi, have presented a review on synthesis of oxazole, oxazoline and isoxazoline derived marine natural products [12]. The current review is concentrates on the diverse biological potential of oxazole derivatives in the new millennium, as no such extensive review article is reported recently.

## Biological activities of oxazole

Pharmacological interventions of oxazole derivatives are voluminous, but this article covers the most relevant ones.

### Antimicrobial activity

Zhang et al. synthesized a chain of some propanoic acid derivatives and examined them for antibacterial and antifungal potential against various strains using different reference drugs as mentioned in Table 1. Compounds **5**, **6** and **7** exhibited most potent antibacterial activities but poor antifungal activity (Table 1) [3].

**Table 1:** Minimal inhibition concentration (µg/ml) of compounds **5**, **6** and **7**

Compd.	MIC (µg/ml)				
	*EC*	*SA*	MRSA	*BS*	*CA*
**5**	3.12	1.56	1.56	3.12	> 200
**6**	3.12	1.56	1.56	3.12	> 200
**7**	6.25	1.56	1.56	1.56	> 200
Ceftazidime	200	0.78	12.5	6.25	–
Cefradine	25	25	50	50	–
Sodium penicillin	0.78	3.12	3.12	< 0.39	–
Ketoconazole	–	–	–	–	< 0.39

*EC*, *Escherichia coli*; *SA*, *Staphylococcus aureus*; MRSA, Methicillin resistant *Staphylococcus aureus*, *BS*, *Bacillus subtilis*; *CA*, *Candida albicans*

A series of pyrazole linked to oxazole-5-one moiety was synthesized and assessed for their antimicrobial potential against *S. aureus*, *E. coli*, *P. aeruginosa* and *C. albicans*. Ampicillin and streptomycin (10 and 25 µg/ml) were used as reference drugs for antibacterial activity and fluconazole, ketaconazole and clotrimazole (10, 20 and 30 µg/ml) were used for antifungal activity. Compound **8** showed highest activity amongst all the synthesized derivatives (Table 2) [13].

**Table 2:** Biological activities of compound **8**

Compd.	Conc.	Inhibition zone (mm) for antimicrobial activity			
		*E. coli*	*P. aeruginosa*	*S. aureus*	*C. albicans*
**8**	15	–	–	–	−
	20	–	–	–	NA
	25	9.4	7.4	8.3	NA
	30	13.7	8.5	10.6	−
	45	NA	NA	NA	+++
	60	NA	NA	NA	+++
Ampicillin	10	10	–	08	−
	25	18	08	13	NA
Streptomycin	10	18	06	8	NA
	25	20	18	9	NA
Fluconazole	10	NA	NA	NA	−
	20	NA	NA	NA	++
	30	NA	NA	NA	++
Ketaconazole	10	NA	NA	NA	−
	20	NA	NA	NA	+
	30	NA	NA	NA	+++
Clotrimazole	10	NA	NA	NA	++
	20	NA	NA	NA	+++
	30	NA	NA	NA	+++

Tanitame et al. prepared a range of novel pyrazole, oxazole and imidazole derivatives and checked for its antibacterial potential against various strains such as *Staphylococcus aureus* FDA 209P, *S. aureus* KMP 9, *Escherichia Coli* NIHJ JC-2 and, *E. coli* W3110 *∆acrA*. Sparfloxacin and novobiocin have been used as reference drugs. Among the tested oxazole derivatives, compound **9** was found to possess maximum antibacterial activity but was less potent as compared to pyrazole and imidazole derivatives (Table 3) [14].

**Table 3:** Minimal inhibition concentration (µg/ml) of compound **9**

Compd.	MIC (µg/ml)			
	*S. aureus*		*E. coli*	
	FDA 209P	KMP 9	NIHJ JC-2	W3110 *∆acrA*
**9**	2	2	64	4
Sparfloxacin	0.125	128	0.032	0.004
Novobiocin	0.25	0.25	64	0.5

Aagalwe et al. carried out the preparation of4-substituted aryl 2–4-disubstituted phenoxy methyl 4-oxazol-5-one derivatives (**10**) and screened their antibacterial potential against *E. coli* and *Xanthomonas citri* using cup-plate method against the standard drug streptomycin. Amongst all the compounds, **10b**, **10c**, **10e**, **10f** showed highest activity against *E. coli* and compounds **10a**, **10b**, **10c**, **10d**, **10e**, **10g** showed highest activity against *X. citri* (Table 4) [15].

**Table 4:** Antibacterial activity data of compound **10**

Compd.	Zone of inhibition (mm)	
	*E. coli*	*X. citri*
**10a**	08	13
**10b**	12	15
**10c**	13	12
**10d**	10	13
**10e**	12	14
**10f**	12	08
**10g**	07	13
Streptomycin	12	14

Ryu et al. performed the synthesis of series of benzo[*d*]oxazoles and evaluated its antifungal potential against various strains using 5-flourocytosine as a reference drug. The activity of compound **11** and **12** was found to be superior or comparable to reference drug (Table 5) [16].

**Table 5:** Antifungal activity of compounds **11** and **12**

Compd.	MIC (µg/ml)					
	*Candida albicans*	*Candida tropicalis*	*Candida krusei*	*Candida neoformans*	*Aspergillus niger*	*Aspergillus flavus*
**11**	1.6	3.2	3.2	1.6	1.6	3.2
**12**	0.8	3.2	3.2	1.6	0.8	1.6
5-Flourocytosine	3.2	3.2	3.2	3.2	1.6	1.6

Singh et al. carried out the synthesis of substituted oxa/thiazoles and evaluated its antibacterial potential against various bacterial strains using the reference drugs ampicillin and ciprofloxacin. Antibacterial activity of the compound **(13)** revealed that **13a** had good activity against *E. coli* (20 mm); **13b**, **13d** and **13e** had equipotent activity as standard compound and **13c** exhibited good antibacterial potential. In case of antibacterial activity of compound **14,** the derivatives **14a**, **14c**, **14d** showed good antibacterial activity and **14b** exhibited better antibacterial activity than standard drugs. Results are presented in Table 6 [17].

**Table 6:** Bacterial growth inhibition of compounds **13** and **14**

Compd.	Bacterial growth inhibition (diameter in mm)			
	*S. aureus*	*E. coli*	*P. vulgaris*	*K. pneumonia*
**13a**	–	20	–	–
**13b**	19	–	–	–
**13c**	23	–	22	–
**13d**	–	–	–	21
**13e**	19	21	–	–
**14a**	–	20	–	21
**14b**	25	–	–	23
**14c**	–	–	22	–
**14d**	20	–	–	21
Ampicillin	20	18	18	15
Ciprofloxacin	20	22	20	21

Kamble et al. synthesized various oxazole-2-amine and its analogues and used *S. aureus* and *E. coli* for examining their antibacterial activity using amoxicillin as standard drug. The compounds, (*E*)-4-(benzofuran-2-yl)-*N*-benzylideneoxazol-2-amine **(15)** and (*E*)-*N*-(4-nitrobenzylidene)-4-(benzofuran-2-yl)oxazol-2-amine (**16**) showed appreciable activity as compared to standard drug (Table 7) [18].

**Table 7:** Antibacterial activity data of compounds **15** and **16**

Compd.	Bacterial growth inhibition in mm	
	*S. aureus*	*E. coli*
**15**	20	17
**16**	18	15
Amoxicillin	30	27

Benzoxazole-5-carboxylatederivatives were prepared and their antimicrobial activity was evaluated by Chilumula et al. against Gram positive and Gram negative bacterial (*S. typhi*, *E.* *coli, S.* *aureus* and *B.* *subtilis*) and fungal strains (*C. albicans* and *A. niger*). The results were evaluated using ampicillin and clotrimazole as a reference drugs for antimicrobial activity. Compound **17** showed the highest activity whereas compound **18** had much higher potency than other tested compounds. Results are mentioned in Table 8 [19].

**Table 8:** Antimicrobial activity data of compounds **17** and **18**

Compd.	Inhibition zone in mm					
	*BS*	*SA*	*EC*	*ST*	*CA*	*AN*
**17**	23	21	20	18	28	20
**18**	24	22	21	20	30	21
Ampicillin	22	20	18	17	–	–
Clotrimazole	–	–	–	–	27	19

*BS*, *Bacillus subtilis*; *SA*, *Staphylococcus aureus*; *EC*, *Escherichia coli*; *ST*, *Salmonella typhi*; *CA*, *Candida albicans*; *AN*, *Aspergillus niger*

Synthesis of series of heterocyclic derivatives and its antibacterial potential against various organisms such as *B. subtilis*, *S. aureus, E. coli* and *K. pneumonia* using standard drug ampicillin was done by Kaspady et al. 2-*tert*-Butyl-4-(4-chlorophenyl)oxazole (**19**) and 4-(4-bromophenyl)-2-*tert*-butyloxazole (**20**) were found to be the most active compounds (Table 9) [20].

**Table 9:** Zone of inhibition in mm of compound **19** and **20**

Compd.	*B. subtilis*	*S. aureus*	*E. coli*	*K. pneumonia*
**19**	***	***	**	**
**20**	***	***	***	**
Ampicillin	*****	*****	*****	*****

* Less than 12 mm; **12–15 mm; ***15–21 mm; ****21–27 mm; *****> 27 mm

Shamsuzzaman et al. synthesized a series of 2ˈ-amino-5α-cholest-6-eno [6,5-d] oxazole derivatives (**21**). Disk diffusion assay was used to examine the antimicrobial activity using various bacterial and fungal strains against chloramphenicol and nystatin which were used as reference drugs for the study. Out of all the compounds, **21b** was found to be the most active one. Results are presented in Tables 10 and 11 [21].

**Table 10:** Antifungal activity of synthesized derivatives

Compd.	Inhibition zone (mm) at 100 µg/ml				
	*Ca*	*Cg*	*Psp*	*Fo*	*An*
**21a**	20.1 ± 0.2	10.1 ± 0.2	15.1 ± 0.2	12.1 ± 0.2	11.2 ± 0.5
**21b**	21.5 ± 0.5	15.2 ± 0.5	16.2 ± 0.5	13.1 ± 0.5	12.5 ± 0.2
**21c**	19.1 ± 0.5	09.2 ± 0.2	14.5 ± 0.2	10.1 ± 0.2	10.1 ± 0.5
Nystatin	29.0 ± 0.5	29.0 ± 0.5	24.5 ± 0.5	19.5 ± 0.5	19.5 ± 0.5

*Ca*, *Candida albicans*; *Cg*, *Candida glabrata*; *Psp*, *Penicillium* spp.; *Fo*, *Fusarium oxyporium*; *An*, *Aspergillus niger*

**Table 11:** Antibacterial activity of synthesized derivatives

Compd.	Inhibition zone (mm) at 100 µg/ml					
	*Bs*	*Sp*	*Sa*	*Pa*	*St*	*Ec*
**21a**	32	128	128	64	128	128
**21b**	64	128	64	64	64	128
**21c**	128	256	128	64	128	256
Chloramphenicol	32	32	32	32	32	32

*Bs*, *Bacillus subtilis*; *Sp*, *Streptococcus pyogenes*; *Sa*, *Staphylococcus aureus*; *Pa*, *Pseudomonas aeruginosa*; *Ec*, *Escherichia coli*; *St*, *Salmonella typhimurium*

Tomi et al. synthesized new derivatives of five membered heterocyclic compounds containing oxazole and benzothiazole rings and then screened them for their antimicrobial activity using ofloxacin and ketoconazole as standard drugs. Amongst the tested oxazole derivatives (**22**), three compounds, **22a**, **22b**, **22c** came out to be active against bacterial and fungal strains (Table 12) [22].

**Table 12:** Antimicrobial activity of oxazole derivatives

Compd.	N	Inhibition zone in mm				
		*E. coli*	*S. aureus*	*P. aeruginosa*	*A. niger*	*C. albicans*
**22a**	4	12	9	11	10	12
**22b**	7	11	8	11	9	16
**22c**	8	12	9	13	11	13
Ofloxacin	–	17	16	16	–	–
Ketoconazole	–	–	–	–	20	30

A chain of 1,3-oxazole derivatives was prepared and examined for microbial inhibition potential against various bacterial and fungal strains by Sadek et al. Ofloxacin and ketoconazole were used as reference drugs for antimicrobial study. The 1,3oxazole derivative (**23**) showed notable activity at higher concentration (200 µg/ml) (Table 13) [23].

**Table 13:** Antimicrobial activity of compound **23**

Compd.	MIC in µg/ml		
	*S. aureus*	*E. coli*	*A. niger*
**23**	200	200	200
Ofloxacin	10	12.5	–
Ketoconazole	–	–	12.5

Synthesis of a number of multi-substituted oxazoles containing a heterocyclic moiety was carried out and checked for antibacterial activity by Babulreddy et al. against different bacterial strains (*S. aureus, E. coli*, *B. subtilis, K. pneumonia)*. Ampicillin was used as reference drug for antibacterial activity. Out of all the derivatives investigated, **24**, **25, 26** and **27** showed pronounced antibacterial activity whose results are mentioned in Table 14 [24].

**Table 14:** Antibacterial activity of multi-substituted oxazoles

Compd.	Inhibition zone (MIC in µg/ml)			
	*B. subtilis*	*S. aureus*	*E. coli*	*K. pneumonia*
**24**	+++ (258)	++++ (294)	+++ (276)	+++ (266)
**25**	++++ (264)	++++ (298)	+++ (254)	++ (277)
**26**	++++ (255)	++++ (312)	+++ (284)	++++ (291)
**27**	++++ (310)	++++ (285)	++++ (289)	++++ (273)
Ampicillin	+++++ (3.28)	+++++ (3.36)	+++++ (3.88)	+++++ (4.00)

Dabholkar et al. carried out the synthesis of 2, 4-disubstituted oxazoles and checked their antibacterial activity against Gram negative bacteria, *E. coli* and *P. aeruginosa* and Gram-positive bacteria *S. aureus* and *C.* *diphtheriae.* Ampicillin trihydrate was the standard drug used and inhibition zone was measured in mm. Compound **28** showed convincing activity against the various bacterial strains. Results are presented in Table 15 [25].

**Table 15:** Antibacterial activity of compounds **28a** and **28b**

Compd.	Zone of inhibition (in mm)			
	*S. aureus*	*C. diphtheriae*	*P. aeruginosa*	*E. coli*
**28a**	13	16	18	14
**28b**	14	18	18	15
Ampicillin trihydrate	26	28	24	21

Some new aryl oxazoles were prepared by Dawood et al. and then assessed its antimicrobial potential. Reference drugs used were chloramphenicol and fluconazole. Compound **29** was found to have the highest antibacterial and antifungal activity (Table 16) [26].

**Table 16:** Minimum inhibitory concentration of compound **29**

Compd.	MIC in µg/ml							
	*E.c*	*S.a*	*B.s*	*P.a*	*S.r*	*A.f*	*C.a*	*G.c*
**29**	250	31.25	125	62.5	125	31.25	62.5	62.5
Chloramphenicol	15.60	31.25	31.25	31.25	–	–	–	–
Fluconazole	–	–	–	–	250	125	250	250

*E.c*, *Escherichia coli*; *S.a*, *Staphylococcus aureus*; *B.s*, *Bacillissubtillis*; *P.a*, *Pseudomonas aeruginosa*; *S.r*, *Syncephalastrumracemosum*; *A.f*, *Aspergillusfumigatus*; *C.a*, *Candidaalbicans*; *G.c*, *Geotrichumcandidum*

Synthesis of a chain of oxazole derivatives was done by Singh et al. and were checked for its antimicrobial potential and compared with reference drugs ciprofloxacin, gatifloxacin, fluconazole. Among the tested compounds, 3-(2-(4-methoxybenzylideneamino)oxazol-4-ylamino)-2*H*-chromen-2-one (**30**) showed potent antibacterial activity, 3-(2-(2-hydroxybenzylideneamino)oxazol-4-ylamino)-2*H*-chromen-2-one (**31**) exhibited moderate antifungal activity, 3-chloro-4-(4-methoxyphenyl)-1-(4-(2-oxo-2*H*-chromen-3-ylamino)oxazol-2-yl)azetidin-2-one (**32**) showed potent antibacterial activity, and 3-chloro-4-(2-hydroxyphenyl)-1-(4-(2-oxo-2*H*-chromen-3-ylamino)oxazol-2-yl)azetidin-2-one (**33**) exhibited most potent antifungal activity. Results are mentioned in Table 17 [27].

**Table 17:** Antimicrobial activity of compounds **30**, **31, 32** and **33**

Compd.	Bacterial growth inhibition (mm)				Fungal growth inhibition (mm)
	*S. aureus*	*E. coli*	*P. vulgaris*	*K. pneumoniae*	*C. albicans*
**30**	19	22	16	20	8
**31**	14	–	12	18	16
**32**	28	30	21	22	–
**33**	–	9	–	–	30
Ciprofloxacin	20	22	20	20	–
Gatifloxacin	25	22	20	20	–
Fluconazole	–	–	–	–	29

Taile et al. prepared a series of oxazol-5-ones and screened its antibacterial potential against various pathogenic bacteria using ciprofloxacin and sulphacetamide as reference drugs. The prepared derivatives were also examined for their antifungal potential against *Aspergillus niger* and *Candida albicans*. The zone of inhibition was checked in comparison with gentamycin and clotrimazole. Compounds **34** and **35** exhibited good antibacterial activity whereas the compounds **36** and **37** showed good antifungal activity. Results are given in Table 18 [28].

**Table 18:** Antimicrobial activity of compounds **34**, **35**, **36** and **37**

Compd.	Diameter of Bacterial growth inhibition				Diameter of Fungal growth inhibition	
	*SA*	*BS*	*EC*	*KA*	*CA*	*AN*
**34**	29	28	24	18	16	24
**35**	30	26	29	22	17	17
**36**	19	24	16	17	21	22
**37**	23	15	23	19	22	21
Ciprofloxacin	34	29	35	22	–	–
Sulphacetamide	31	26	29	21	–	–
Gentamycin	–	–	–	–	21	25
Clotrimazole	–	–	–	–	23	24

*SA*, *Staphylococcus aureus*; *BS*, *Bacillus subtilis*; *EC*, *Escherichia coli*; *KA*, *Klebsiella aerogenes*; *CA*, *Candida albicans*; *AN*, *Aspergillus niger*

Prasad et al. carried out the synthesis of compounds **38** and **39** and evaluated their antimicrobial activity by disk diffusion method against various bacterial strains using ciprofloxacin and ketoconazole as reference drugs. Both the derivatives exhibited good antimicrobial activity and the results are presented in Table 19 [29].

**Table 19:** Antimicrobial data of the compounds **38** and **39**

Compd.	Zone of inhibition (mm) by disk diffusion method					
	*SA*	*BC*	*EC*	*PA*	*AN*	*AF*
**38**	24	25	28	27	27	27
**39**	25	24	24	28	24	25
Ciprofloxacin	38	39	40	40	–	–
Ketoconazole	–	–	–	–	40	39

*SA*, *Staphylococcus aureus*; *BC*, *Bacillus cereus*, *PA*, *Pseudomonas aeruginosa*; *EC*, *Escherichia coli*; *AN*, *Asperigillusniger*; *AF*, *Aspergillus fumigates*

Various oxazole derivatives were prepared and assessed for their antimicrobial potential by Patel et al. against various Gram positive (*S. aureus* and *S. pyogenes*), Gram negative (*P. aeruginosa* and *E. coli*) and fungal strains (*C. albicans, A. niger* and *A. clavatus*). Ampicillin, chloramphenicol, ciprofloxacin, nystatin and griseofulvin have been used as reference drugs. Compound **40** was found to be most potent antibacterial agent whereas compound **41** was the most potent antifungal agent (Table 20) [30].

**Table 20:** Minimum inhibitory concentration for compounds **40** and **41**

Compd.	MIC in µg/ml						
	*Ec*	*Pa*	*Sa*	*Sp*	*An*	*Af*	*Ac*
**40**	50	100	50	250	1000	> 1000	> 1000
**41**	200	500	200	200	500	500	500
Ampicillin	100	100	250	100	–	–	–
Chloramphenicol	50	50	50	50	–	–	–
Ciprofloxacin	25	25	50	50	–	–	–
Nystatin	–	–	–	–	100	100	100
Griseofulvin	–	–	–	–	500	100	100

*Ec*, *Escherichia Coli*; *Pa*, *Pseudomonas aeruginosa*; *Sa*, *Staphylococcus aureus*; *Sp*, *Streptococcus pyogenes*; *Ca*, *Candida albicans*; *An*, *Aspergillus niger*; *Ac*, *Aspergillus clavatus*

Anand et al. synthesized various substituted benzoxazoles and evaluated their antimicrobial potential against *S. aureus, E. coli, C. albicans* and *C. glabrata* using trimethoprim and miconazole as standard drug. Among the investigated compounds, 2-methoxy-5-chlorobenzo[*d*]oxazole (**42**) and 2-ethoxybenzo[*d*]oxazole (**43**) had excellent antibacterial activity whereas 2-ethoxy-5-chlorobenzo[*d*]oxazole (**44**) and 2-methoxybenzo[*d*]oxazole (**45**) had excellent antifungal activity (Table 21) [31].

**Table 21:** Antimicrobial activity of compounds **42**, **43**, **44** and **45**

Compd.	Zone of inhibition (mm)			
	*SA*	*EC*	*CA*	*CG*
**42**	18	16	19	16
**43**	18	15	14	16
**44**	17	14	19	18
**45**	16	15	18	20
Trimethoprim	25	23	–	–
Miconazole	–	–	26	15

*SA*, *Staphylococcus aureus*; *EC*, *Escherichia coli*; *CA*, *Candida albicans*; *CG*, *Candida glabrata*

Patel et al. synthesized a series of 2-[2-(2,6-dichloro-phenylamino)-phenyl methyl]-3-{4-[(substituted phenyl) amino]-1,3-oxazol-2-yl-}quinazolin-4(3*H*)ones and examined its antibacterial potential against *S. aureus* and *S. pyogenes*, *P. aeruginosa* and *E. coli* and *C. albicans*, *A. niger* and *A. clavatus* using chloramphenicol, gentamycin, ampicillin, ciprofloxacin and norfloxacin as reference drugs for antibacterial activity and nystatin and griseofulvin for antifungal activity. 2-(2-(2,6-Dichlorophenylamino)benzyl)-3-(4-(2-chlorophenylamino)oxazol-2-yl)quinazolin-4(3*H*)-one (**46**) was found to possess good activity against all the bacterial strains and *Candida albicans* but not against *Aspergillus niger* and *Aspergillus clavatus* whereas 2-(2-(2,6-dichlorophenylamino)benzyl)-3-(4-(phenylamino)oxazol-2-yl)quinazolin-4(3*H*)-one (**47**) was found to be active against *Aspergillus niger* and *Aspergillus clavatus.* Results of antimicrobial study are shown in Table 22 [32].

**Table 22:** Antimicrobial activities of the compounds **46** and **47**

Compd.	MIC (µg/ml)						
	*E. coli*	*P. aeruginosa*	*S. aureus*	*S. pyogenes*	*C. albicans*	*A. niger*	*A. clavatus*
**46**	100	100	100	100	500	1000	500
**47**	100	1000	1000	500	100	100	100
Gen	0.05	1	0.25	0.5	–	–	–
Amp	100	100	250	100	–	–	–
Chlorl	50	50	50	50	–	–	–
Cipro	25	25	50	50	–	–	–
Nor	10	10	10	10	–	–	–
Nys	–	–	–	–	100	100	100
Gri	–	–	–	–	500	100	100

*Gen* Gentamycin, *Amp* Ampicillin, *Chlor* Chloramphenicol, *Cipro* Ciprofloxacin, *Nor* Norfloxacin, *Nys* Nystatin, *Gri* Griseofulvin

Padmavathi et al. synthesized a new class of amido linked bis heterocycles and checked them for antibacterial and antifungal activity against *S. aureus, B. subtilis*, *P. aeruginosa*, *K. pneumonia*, *A. niger* and *P. chrysogenum* using chloramphenicol and ketoconazole as standard drugs. Among the prepared oxazole derivatives, **48** was found to possess most effective antimicrobial activity at 100 µg/ml (Table 23) [33].

**Table 23:** Antibacterial and antifungal potential of the compound **48**

Compd.	Inhibition zone in mm					
	*S. aureus*	*B. subtilis*	*P. aeruginosa*	*K. pneumoniae*	*A. niger*	*P. chrysogenum*
**48**	23	22	21	24	27	29
Std.	35*	38*	30*	42*	–	–
Std	–	–	–	–	36**	38**

Std. Chloramphenicol*; Ketoconazole**

A series of new oxazole derivatives were prepared and assayed for their antibacterial activity against Gram-positive bacteria and Gram-negative bacteria by Reddy et al. using penicillin and streptomycin as reference drugs. The compounds **49** and **50** were found to possess good antibacterial activity as compared to standard drugs. Results are shown in Table 24 [34].

**Table 24:** Antibacterial activity of the compound **49** and **50**

Compd.	Minimum inhibitory concentration in µg/ml					
	*BS*	*BSph*	*SA*	*PA*	*KA*	*CV*
**49**	7 ± 0.7	8 ± 0.4	10 ± 0.4	8 ± 0.4	8 ± 0.5	16 ± 0.3
**50**	8 ± 0.4	8 ± 0.4	9 ± 0.4	10 ± 0.4	12 ± 0.8	20 ± 0.8
Penicillin	10 ± 0.5	19 ± 0.8	16 ± 0.8	18 ± 0.5	20 ± 1.0	18 ± 0.3
Streptomycin	10 ± 0.6	14 ± 0.9	14 ± 1.1	18 ± 1.0	20 ± 0.8	16 ± 1.2

*BS*, *Bacillus subtilis*; *BSph*, *Bacillus sphaericus*; *SA*, *Staphylococcus aureus*; *PA*, *Pseudomonas aeruginosa*; *KA*, *Klebsiella aerogenes*; *CV*, *Chromobacterium violaceum*

Several new spiroindoline-based heterocycles were made by Rahman et al. and examined for their antimicrobial potential. Among the tested derivatives, compound **51** was found to be the most effective against *Bacillus subtilis*, *Bacillus megatherium*, *E. coli*, *Aspergillus niger* and *Aspergillus oryzae*. Ampicillin, chloramphenicol and fluconazole were used as reference drugs (Table 25) [35].

**Table 25:** Inhibition zone (in mm) of new spiroindoline-based heterocycles

Compd.	Inhibition zone (in mm)				
	*B. subtilis*	*B. megatherium*	*E. coli*	*A. niger*	*A. oryzae*
**51**	87	86	45	80	86
Ampicillin	41	29	26	33	–
Chloramphenicol	28	55	48	35	–
Fluconazole	–	–	–	22	16

**Fig. 2:**
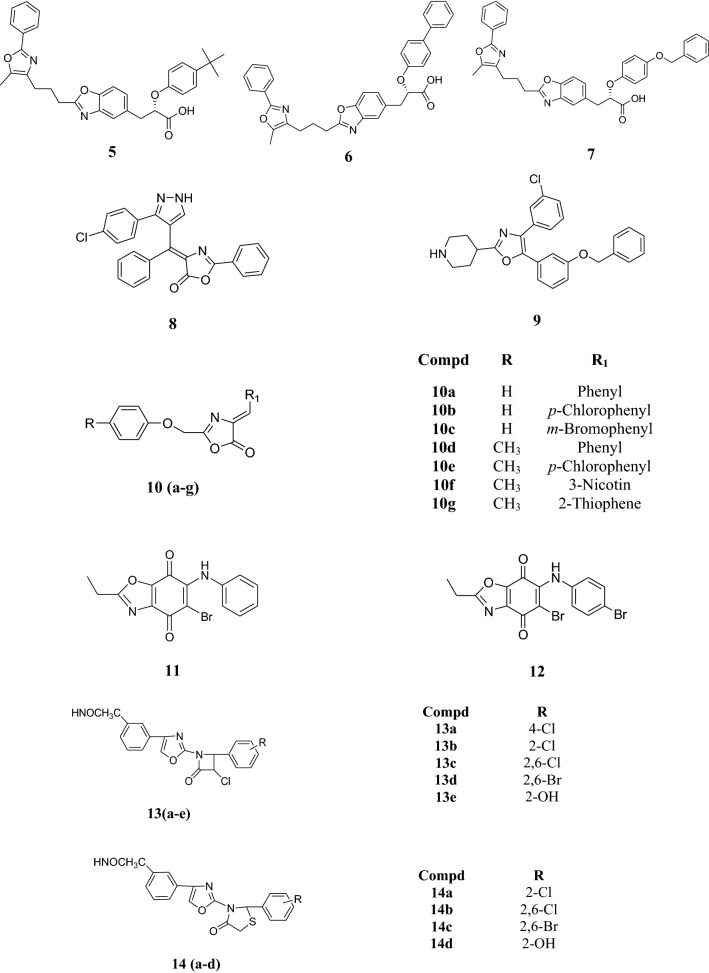
Structures of the most active antimicrobial compounds

**Fig. 3:**
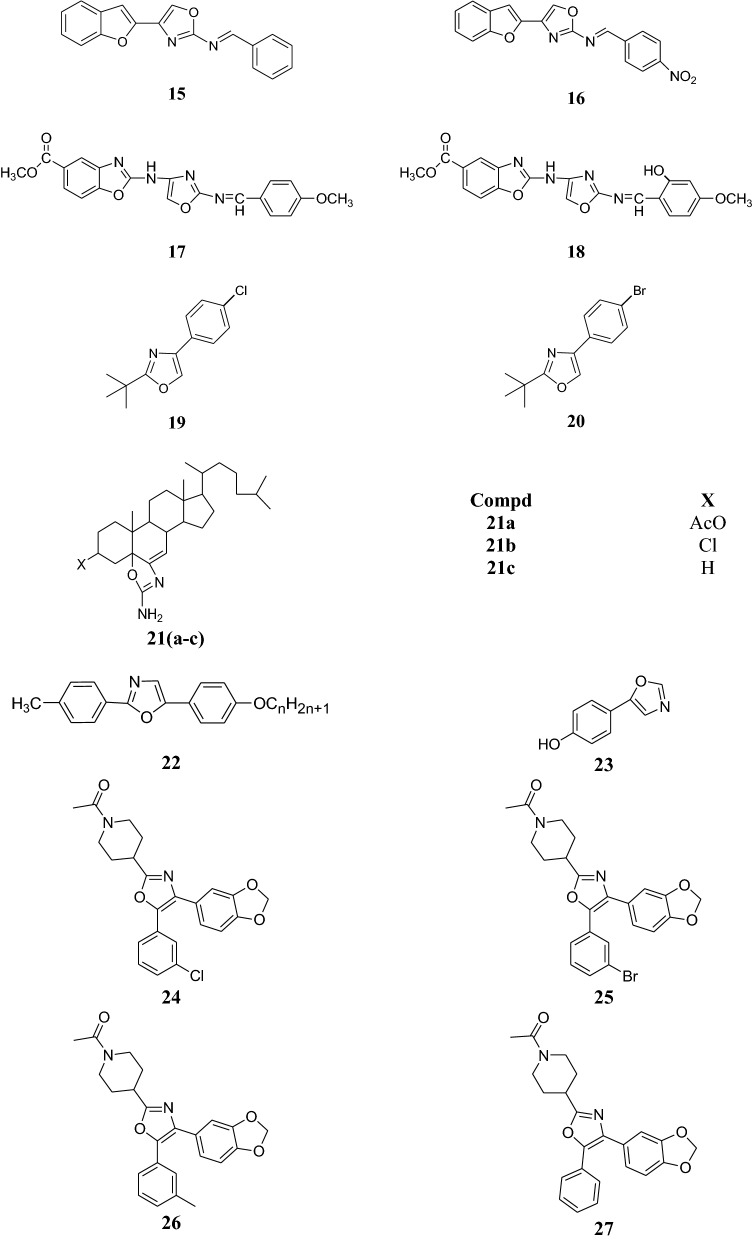
Structures of the most active antimicrobial compounds

**Fig. 4:**
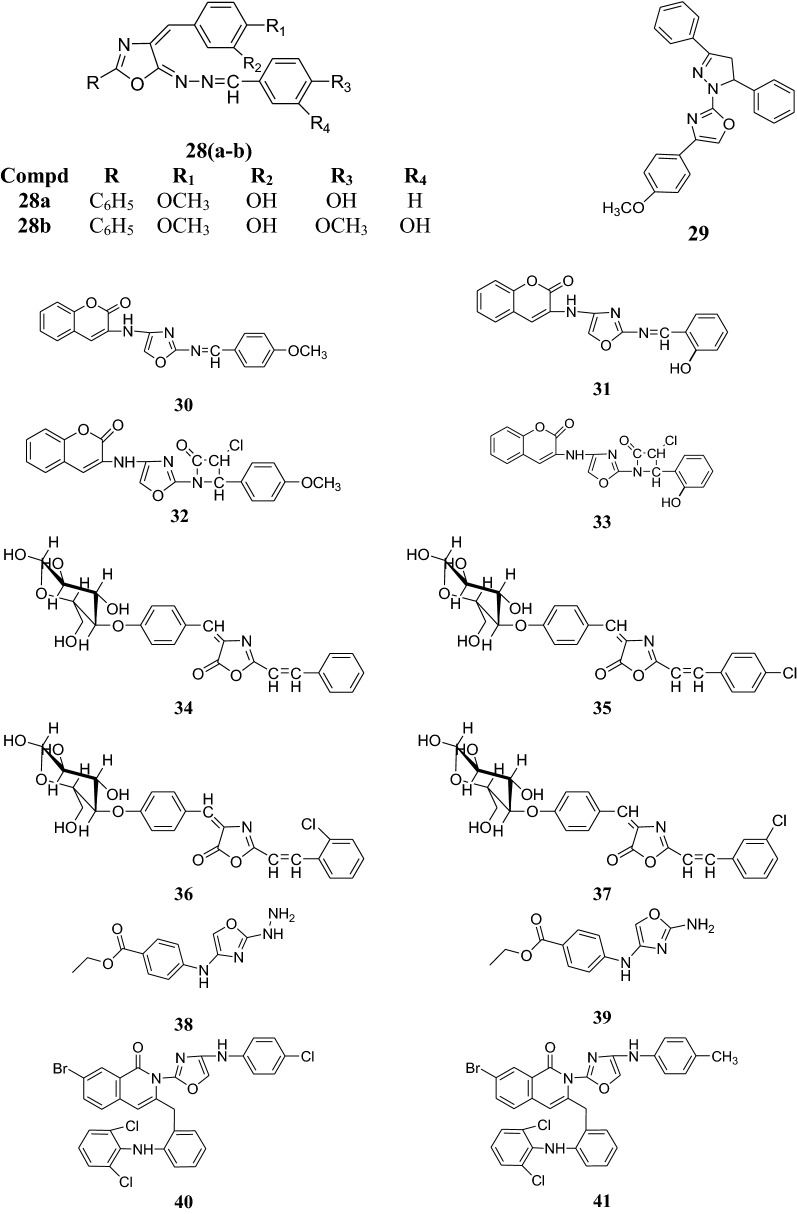
Structures of the most active antimicrobial compounds

**Fig. 5:**
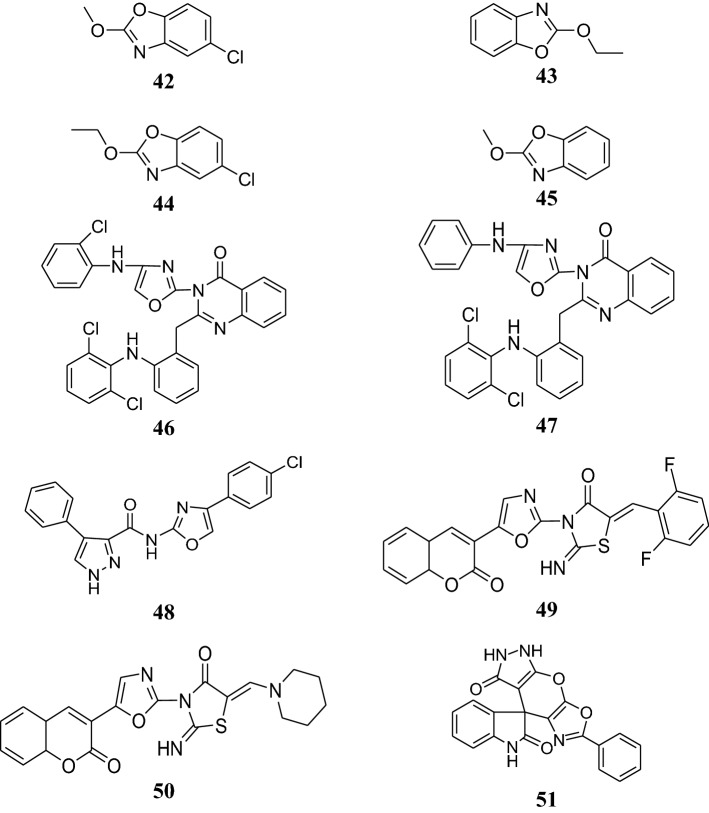
Structures of the most active antimicrobial compounds

The structures of the most active antimicrobial compounds (**5**–**51**) are shown in Figs. 2, 3, 4, 5.

### Anticancer activity

Cantalejo et al. synthesized bisoxazoles and evaluated their anticancer activity against the cancer cell line HT-29. As well as tested in an ex vivo system using recombinant human choline kinase (ChoK) to assess the inhibitory potency of the derivatives towards ChoK. Compound **52** was found to possess the maximum antiproliferative activity with an IC_50_ value of 0.84 ± 0.005 whereas compound **53** was found to be most active in case of ex vivo study (IC_50_ = 0.30 ± 0.003) [36].

The molecular interactions of three ruthenium complexes were studied by Barca et al. in isolated mammalian nuclei. The complexes were chemotherapeutic agents that are effective in reducing metastatic tumours in vivo and were compared with antitumour drug *cis*-diamminedichloroplatinum (CDDP) (**57**). Na *trans*-RuCl_4_ (DMSO) imidazole (NAMI) (**54**), Na *trans*-RuCl_4_ (DMSO) oxazole (NAOX) (**55**) and Na *trans*-RuCl_4_(TMSO) isoquinoline (TEQU) (**56**) were the complexes under investigation. The Ru complexes were screened for toxicity on V79 cells which showed that NAMI and NAOX did not reduce the cloning efficiency, only TEQU reduced the cloning efficiency as well as induced a number of mutants in V79 cells in culture [37].

Kumar et al. carried out the synthesis of a series of oxazole derivatives and evaluated its antitumour activity using various cell lines. Among all the screened derivatives, compounds **58** and **59** were found to have potent cytotoxic action against tested cell lines (Table 26) [4].

**Table 26:** Cytotoxicity profile of compounds **58** and **59**

Compd.	Cancer cell lines					
	PC3	DU145	LnCaP	MCF7	MDA231	PaCa2
**58**	42.8	31.8	59.8	28	90.4	40.6
**59**	349.8	80.5	181.6	14.1	216.3	26

Liu et al. carried out the preparation of various trisubstituted oxazole derivatives and checked their antitumour potential against two cancer cells, PC-3 (human prostate cancer) and A431(human epidermoid carcinoma)using 5-flourouracil as reference. Among the investigated compounds, **60**, **61** and **62** were the most effective (Table 27) [38].

**Table 27:** Antiproliferative potential of the synthesized derivatives

Compd.	IC_50_ (µM)	
	PC-3	A431
**60**	0.0030	0.0031
**61**	0.0047	0.0076
**62**	0.0035	0.0026
5 Flouro-uracil	0.016	0.018

Mahal et al. studied the antitumoral properties of a metabolite of the South-African bush willow *Combretum caffrum*, *cis*-stilbene combretastatin A-4 (CA-4). However the conversion of CA-4 into the *trans*-isomer and its poor solubility limits its use in anticancer therapy. In order to overcome these drawbacks different heterocycles were integrated with CA-4 which led to the formation of CA-4 analogues having imidazole and oxazole rings. The halogen substituted oxazoles showed enhanced anticancer activity and showed antivascular activity as well. Different cell lines used were human HT-29 colon carcinoma, human 518A2 melanoma and Ea.hy926 endothelial hybrid cells. The oxazole derivatives **63** (**a**–**c**) were found to be active whose IC_50_ values are given in Table 28 [39].

**Table 28:** Cytotoxicity profile of compound **63**

Compd.	IC_50_ (nM)		
	HT-29	518A2	Ea.hy926
**63a**	6 ± 1	3 ± 2	9 ± 1
**63b**	11 ± 1	2 ± 1	31 ± 3
**63c**	76 ± 3	50 ± 15	77 ± 4

Pilch et al. characterized two synthetic hexaoxazole-containing macrocyclic compounds, HXLV-AC (**64**) and HXDV (**65**) and evaluated its antiproliferative potential against various cell lines. Cytotoxicity was evaluated using MTT assay and the IC_50_ values are shown in Table 29 [40].

**Table 29:** Cytotoxicity of HXDV and HXLV-AC

Compd.	IC_50_ (µM)	
	RPMI 8402	KB3-1
HXLV-AC	0.8 ± 0.3	0.9 ± 0.2
HXDV	0.4 ± 0.1	0.4 ± 0.1

Ohnmacht et al. reported some bisoxazole derivatives and evaluated them for anticancer potential. The analogue **66** was found to be the most effective in the series having high selectivity for the HSP90A over HSP90B quadruplexes. The compound **66** was evaluated for anticancer activity against various cell lines and the IC_50_ values are mentioned in Table 30 [41].

**Table 30:** Cytotoxicity of compound **66**

Cancer cell lines	IC_50_in µmol
A549	1.02
MCF7	1.32
RCC4	0.94
786-o	1.33
Mia-Pa-Ca2	1.25
W138	2.59

Various new oxazole derivatives were synthesized and examined for their antitumour activity by Sączewski et al. Among the synthesized derivatives, compounds **67** and **68** were evaluated against a number of different cell lines using nitrofurantoin, cisplatin, melphalan and thiotepa as reference drugs and the results are mentioned in Table 31 [42].

**Table 31:** IC_50_ values (µM) in human cancer cell lines

Compd.	RT-4	RT-112	5637	KYSE-70	KYSE-510	DAN-G	SISO	LCLC-103H	MCF-7	A-427
**67**	6.57	3.88	3.91	5.30	22.63	12.62	14.12	12.06	5.69	2.33
**68**	3.98	1.41	1.65	2.91	7.00	3.00	2.86	1.33	2.87	1.13
NTF	7.00	Nf	21.3	22.8	29.0	6.74	7.27	2.34	4.44	1.86
CP	1.61	1.22	0.35	0.63	0.44	0.73	0.24	0.90	1.38	1.96
Mph	14.25	4.69	0.31	16.16	8.18	2.65	1.00	4.00	3.71	5.13
Ttp	18.27	3.40	2.0	5.40	4.31	1.66	1.40	6.97	3.23	1.58

*nf* not found, *NTF* Nitrofurantoin, *CP* Cisplatin, *Mph* Melphalan, *Ttp* Thiotepa

Savariz et al. prepared a range ofoxazol-5-one derivatives and carried out the in vitro antitumor evaluation. Doxorubicin was used as a positive control. Among all the synthesized compounds, **69** was found to possess maximum activity against prostate (PC-3) and ovarian (OVCAR-03) cancer cell lines with IC_50_values of 1.50 and 1.07 µM respectively [43].

Three series of novel oxo-heterocyclic fused naphthalimide derivatives were made by Tan et al. and were evaluated for antiproliferative potential using various tumor cell lines. Among the synthesized oxazole derivatives, **70** and **71** were found to be the most active ones (Table 32) [44].

**Table 32:** IC_50_ (µM) of active compounds **70** and **71**

Compd.	A549 (Human lung cancer cell)	P388 (Murine Leukemia Cell)	LO2 (Human Liver Cell)
**70**	0.53	2.50	3.0
**71**	0.89	1.30	1.9
Amonafide	1.10	0.20	5.0

Biersack et al. reported that oxazole-linked combretastatin A-4 analogues (possessing anti-vascular and anti-angiogenic activity) when linked to Ru(η^6^-arene) complex fragments shows additional cytotoxic activity. MTT tests with the oxazoles and their ruthenium complexes revealed them to be effective against cells of human518A2 melanoma and HL-60 leukaemia. Compound **72** showed the highest activity [45].

Hernández et al. did the synthesis of several analogues of the cytotoxic thiopeptide IB-01211 or mechercharmycin A. The cytotoxicity of synthesized analogues was checked against three human tumour cell lines. The peptide heterocycles **73** and **74** were found to be the most active ones (Table 33) [46].

**Table 33:** In vitro cytotoxicity of peptide derivatives

Compd.	Cytotoxicity (GI_50_, µM)		
	A-549 lung carcinoma NSCL	HT-29 colon carcinoma	MDA-MB-231 231breast adenocarcinoma
**73**	0.17	0.12	0.10
**74**	0.12	0.13	0.12

A series of oxazole derivatives were prepared by Lin et al. and the EGFR and Src inhibition activities were checked using gefitinib as reference compound. In vitro cell cytotoxicity of the synthesized derivatives was evaluated against KB and A498 cells using MTT assay. Among all the screened compounds, **75** was found to be the most effective with IC_50_values 0.82 and 3.0 µM against KB and A498 cells respectively [47].

**Fig. 6:**
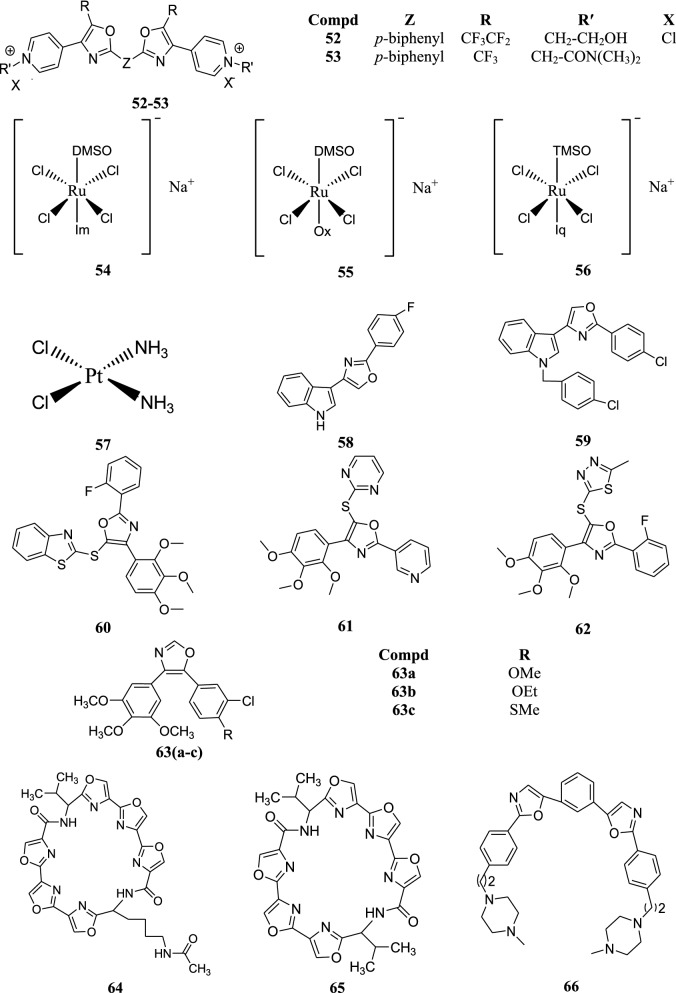
Structures of the most active anticancer compounds

**Fig. 7:**
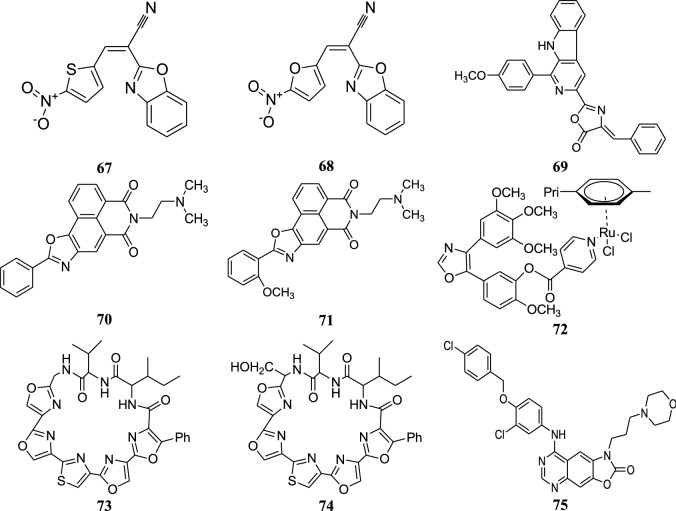
Structures of the most active anticancer compounds

The structures of the most active anticancer compounds (**52**–**75**) are shown in Fig. 6, 7.

### Antitubercular activity

Texaline is an antitubercular oxazole-containing alkaloid which is obtained from *Amyris texana* and *Amyris elemifera*. Several analogues of it, namely 2-(3´-pyridyl)-5-phenyloxazole (**76**) and 2,5-diphenyloxazole (**77**) were synthesized and checked for their antimycobacterial activity by Giddens et al. Both the compounds were found to be effective antitubercular agents. Results are shown in Table 34 [48].

**Table 34:** Antimycobacterial activity of compounds **76** and **77**

Compd.	MIC (µg/ml) for *M. tuberculosis* H_37_Rv	
	MABA	Microbroth
**76**	30.1	31.25
**77**	29.0	31.25

Moraski et al. carried out the synthesis of several oxazoline- and oxazole-containing compounds, which were tested for inhibition of *Mycobacterium tuberculosis* H_37_Rv in two different culture media, GAS and GAST using rifampicin as a positive control. Tween 80 is present in GAST but not in GAS whereas GAST is more iron deficient medium than GAS. Among all the synthesized oxazole derivatives, **78** and **79** were found to be most potent against M*tb*H_37_Rv whose results are presented in Table 35 [5].

**Table 35:** Anti tubercular activity of compound **78** and **79**

Compd.	MIC for *M. tuberculosis* H_37_Rv	
	GAS (µM)	GAST (µM)
**78**	0.47	0.49
**79**	0.73	1.69

Moraski et al. reported various classes of compound sand their antitubercular potential was evaluated against M*tb*H_37_Rv. Among the investigated oxazole derivatives, benzyl 2-phenyloxazole-4-carboxylate (**80**) was found to possess the highest activity against M*tb*H_37_Rv with MIC value of 5.7 ± 2.3 µM [49].

Moura et al. synthesized a number of naphthoimidazoles and naphthoxazoles and evaluated them against susceptible and rifampicin- and isoniazid-resistant strains of *M. tuberculosis*. The study was carried out using *M. tuberculosis* H_37_Rv, RIFr with a His-526 → Tir mutation in the *rpo*B gene and INH^R^ with a Ser-315 → Tir mutation in the *kat*G gene. Among the synthesized naphthoxazoles, compound **81** came out to be most potent. MIC (minimum inhibitory concentration) of the compound **81** against *M. tuberculosis* H_37_Rv, rifampicin-resistant *M. tuberculosis* (RIFr) and isoniazid resistant *M. tuberculosis* (INHr) is given in Table 36 [50].

**Table 36:** MIC values for compound **81**

Compd.	MIC (µg/ml)		
	H_37_Rv	RIFr	INHr
**81**	6.25	1.56	3.12
Rifampicin	≤ 0.125	> 4	≤ 0.125
Isoniazid	≤ 0.06	≤ 0.06	1

Lu et al. carried out the synthesis of a series of substituted thiazole, oxazole and imidazole derivatives. The derivatives were examined for in vitro antitubercular potential using *M. tuberculosis*, and were also evaluated for antibacterial activities. The results for the antimycobacterial activity of oxazole derivatives **82**, **83** are shown in Table 37 [51].

**Table 37:** *In vitro* antitubercular activities of compound **82** and **83**

Compd.	MABA MIC (µM)
**82**	> 128
**83**	> 128

**Fig. 8:**
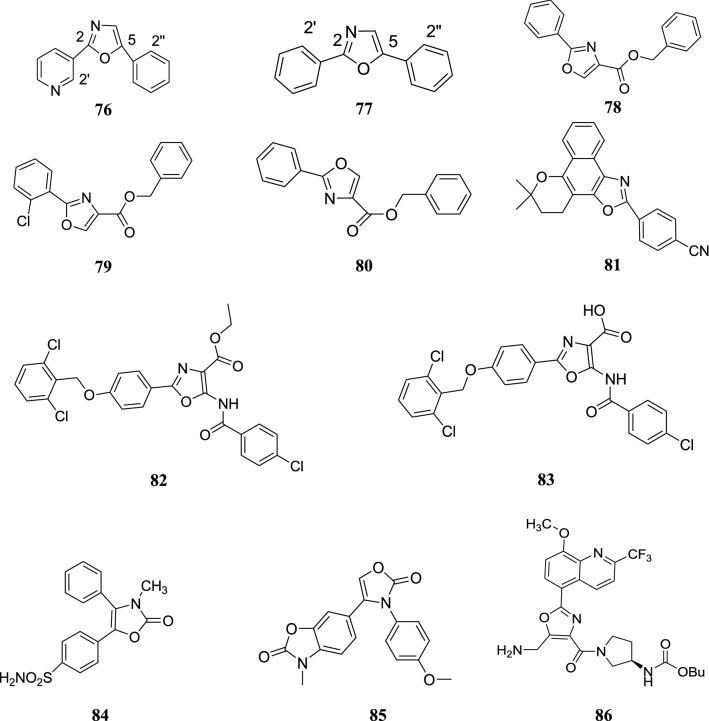
Structures of the most active antitubercular and anti-inflammatory compounds

The structures of the most active antitubercular compounds (**76**–**83**) are shown in Fig. 8.

### Anti-inflammatory activity

Dündar et al. prepared a range of oxazole derivatives and evaluated them for COX-2 inhibition. Homeostasis and gastro protective effects involve COX-1 which is the constitutive form, whereas inflammatory sites involve COX-2. Among the synthesized compounds, **84** was found to possess the highest selective COX-2 inhibition (70.14% ± 1.71) [52].

Eren et al. synthesized a chain of diaryl heterocyclic derivatives and carried out the evaluation of in vitro inhibitory activities against COX-1 and COX-2 isoforms. Among the oxazole derivatives, compound **85** was found to possess the maximum COX-2 inhibition of 47.10% ± 1.05 against the standard drug indomethacin and rofecoxib [6].

Kuang et al. discovered the substituted quinolyl oxazoles as highly effective phosphodiesterase 4 (PDE4) inhibitors. Inflammatory and immune cells involve the expression of PDE4 which is one of the cAMP specific PDE enzymes. Among the investigated compounds, **86** and **87** were found to be most effective with PDE4 IC_50_ values of 1.4 nm and 1 nm, respectively [53].

Kuang et al. carried out the synthesis of series of oxazole derivatives. Among the potent carboxamides, the *N*-benzylcarboxamide was found to exhibit good selectivity for phosphodiesterase 4 over phosphodiesterase 10 and phosphodiesterase 11. Further optimization of this series of potent compounds was carried out which led to the discovery of highly selective PDE4 inhibitors with picomolar potency. Compounds **88**, **89**, **90** and **91** were found to be the most effective PDE4 inhibitors whose IC_50_ values are given in Table 38 [54].

**Table 38:** Anti-inflammatory activity of compounds **88**, **89**, **90** and **91**

Compd.	PDE4 IC_50_ (nm)
**88**	0.05
**89**	0.03
**90**	0.06
**91**	0.04

Perner et al. carried out the synthesis of series of oxazole derivatives and tested for its TRPV1 receptor inhibition. The TRPV1 receptor is responsible for transmission of pain signaling. Among the synthesized compounds, **92** was discovered as a novel TRPV1 antagonist with IC_50_ value of 15 ± 3 nm [55].

Rusch et al. carried out the synthesis of 2-α-keto oxazoles and evaluated them for fatty acid amide hydrolase (FAAH) inhibition. FAAH is a membrane-bound serine hydrolase and is responsible for pain and inflammation. Out of all the tested compounds, **93** was found to be the most effective having an IC_50_ value of 290 nm [56].

Singh et al. prepared some oxazole derivatives and evaluated them for anti-inflammatory potential against carrageenan induced oedema in albino rats. Out of all the screened oxazole derivatives, **94** and **95** were found to be the most potent compounds (Table 39) [57].

**Table 39:** Biological data of compound **94** and **95**

Compd.	Mean increase in paw volume ± SE	Anti-inflammatory activity %	Analgesic activity %
**94**	0.56 ± 0.015	25.3	23.7
**95**	0.49 ± 0.015	27.9	26.3

**Fig. 9:**
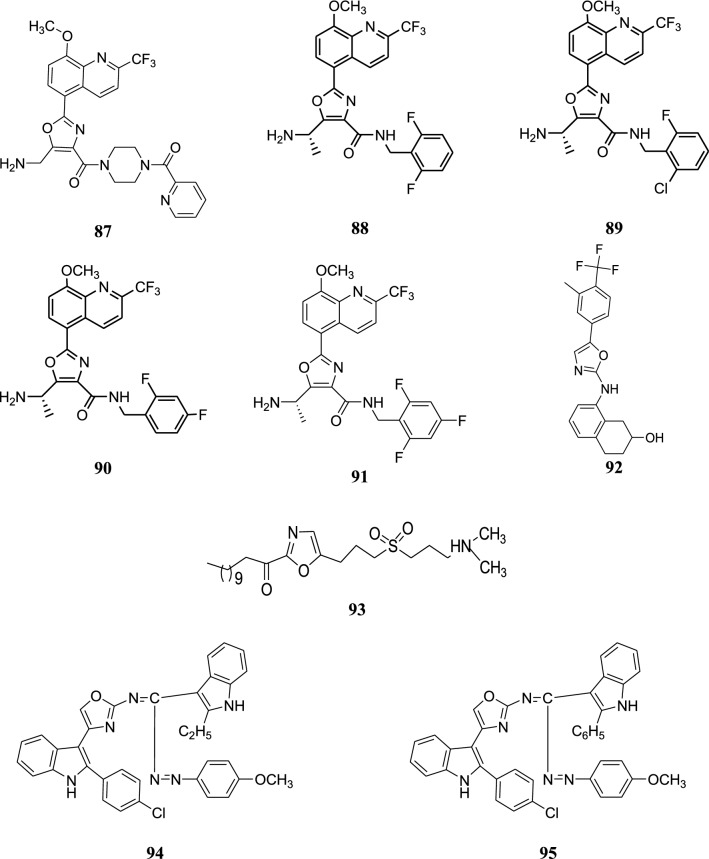
Structures of the most active anti-inflammatory compounds

The structures of the most active anti-inflammatory compounds (**84**–**95**) are shown in Figs. 8 and 9.

### Antidiabetic activity

Ashton et al. synthesized a range of *β*-aminoacylpiperidines with fused five-membered heterocyclic rings (thiazole, oxazole, isoxazole, or pyrazole) as dipeptidyl peptidase IV inhibitors. Out of all the screened oxazole derivatives, (*R*)-3-amino-1-(2-cyclopropyl-6,7-dihydrooxazolo[4,5-*c*]pyridin-5(4*H*)-yl)-4-(2,5-difluorophenyl)butan-1-one (**96**) was found to possess considerable DPP-IV inhibition (IC_50_ = 0.18 µM) [7].

A chain of oxazole derivatives were synthesized by Kumar et al. and checked for PTP-1B inhibitory activity. Protein tyrosine phosphatase-1B (PTP-1B) has been found important for the treatment of diabetes and obesity. Out of all compounds, **97** and **98** exhibited the most promising activity (Table 40) [58].

**Table 40:** Biological data of compounds **97** and **98**

Compd.	PTP-1B inhibitory activity (%)
**97**	89.4
**98**	95.0

Pingali et al. designed and synthesized 1,3-dioxane carboxylic acid derivatives and combined this with substituted oxazole and evaluated them for in vitro PPAR agonistic potential and in vivo sugar lowering and lipid lowering efficacy in animal models using rosiglitazone and tesaglitazar as standard compounds. Compound **99** was found to be the most active (EC_50_ = 0.0015 µM) [59].

Raval et al. designed and synthesized novel thiophene substituted oxazole containing α-alkoxy-phenylpropanoic acid derivatives as highly potent PPAR α/γ dual agonists. Peroxisome proliferator-activated receptors (PPARs) play a very important role in metabolic syndrome whose major manifestations are hyperglycemia, dyslipidemia and obesity. Compound **100** was found to be the most efficacious PPAR α/γ dual agonist and showed the glucose reduction of 72% [60].

**Fig. 10:**
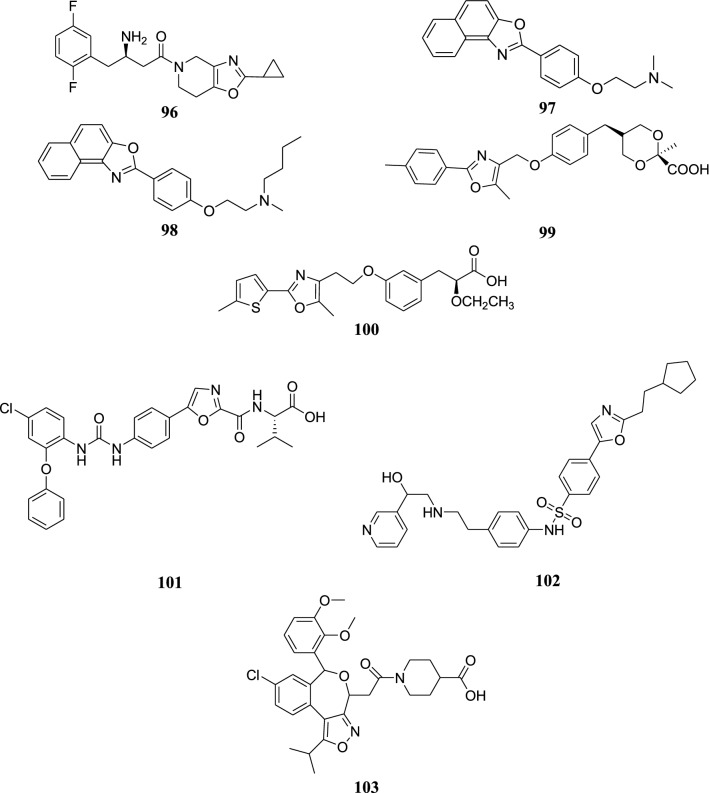
Structures of the most active antidiabetic and antiobesity compounds

The structures of the most active antidiabetic compounds (**96**–**100**) are shown in Fig. 10.

### Antiobesity activity

Jadhav et al. prepared and checked a range of derivative shaving oxazole units for their hDGAT1 inhibition. Diacylglycerol acyltransferase (DGAT1) is an enzyme in obesity which is involved in triglyceride synthesis. Among all the tested oxazole derivatives, **101** was found to possess maximum in vivo plasma triglyceride reduction (91%) [8].

Ok et al. found a range of substituted oxazole derivatives that are effective β3 agonists. Compound **102** was found to be the best β3AR agonist (EC_50_ = 14 nM, 84% activation) [61].

Griebenow et al. prepared a range of novel squalene synthase inhibitors and evaluated them for lipid lowering activity. Squalene synthase is an enzyme which is involved in one of the steps of cholesterol biosynthesis. Compound **103** was found to be most effective. Results are mentioned in Table 41 [62].

**Table 41:** Biological data of compound **103**

Compd.	IC_50_ (nm)	Sterol biosynthesis (%)
**103**	112	79

The structures of the most active antiobesity compounds (**101**–**103**) are shown in Fig. 10.

### Antioxidant activity

Parveen et al. synthesized several 4-arylidene-2-phenyl-5(4*H*)-azlactones and evaluated their antioxidant potential which revealed that compound **104** showed the highest IC_50_ value of 5.15 [9].

### Adrenergic receptor ligand

Drabczyńska et al. prepared a chain of oxazole derivatives and evaluated their affinity at adenosine A_1_ and A_2A_ receptors and anticonvulsant potential. 7-Decyl-1,3-dimethyl-6,7-dihydrooxazolo[3,2-*a*]purine-2,4(1*H*,3*H*)-dione (**105**) was found to possess the maximum affinity towards the A_2A_ receptor but had poor anticonvulsant activity (A_2A_versus[^3^H]MSX-2^b^ % inhibition = 90%) [63].

### Anti progesterone activity

Synthesis of novel oxazole analogs was done by Jin et al. and assessed their antagonist hormonal properties using mifepristone as standard drug. Compounds **106** and **107** showed highly potent antiprogestational activity. Results are mentioned in Table 42 [64].

**Table 42:** Anti-hormonal property of compound **106** and **107**

Compd.	T47D IC_50_ (nM)
**106**	0.34
**107**	0.59
Mifepristone	0.054

### Prostacyclin receptor antagonist

Brescia et al. carried out the synthesis and evaluated the prostacyclin (IP) receptor antagonistic activity of oxazole derivatives. Prostacyclin (PGI_2_), which is an eicosanoid, plays an important role in inhibition of platelet aggregation, vasodilatation, and also acts as an antagonist of thromboxane A_2_. Out of all the tested compounds, **108** was found to be the most effective one. Results are shown in Table 43 [65].

**Table 43:** Biological activity of compound **108**

Compd.	IC_50_ (µM)	
	IPR	HEL cAMP
**108**	0.476 ± 0.193	0.016 ± 0.001

### T-type calcium channel blocker

Lee et al. synthesized a number of oxazole derivatives substituted with arylpipera-zinylalkylamines and biologically evaluated against α_1G_ (Ca_v_3.1) T-type calcium channel. Out of all the synthesized derivatives the most active one was **109** with an IC_50_ value of 0.65 µM, which was found to be comparable with the reference drug mibefradil [66].

### Transthyretin (TTR) amyloid fibril inhibitors

Razavi et al. carried out the synthesis of few oxazole derivatives and assessed as transthyretin (TTR) amyloid fibril inhibitors. 2-(3,5-Dichlorophenyl)-5-ethyloxazole-4-carboxylic acid (**110**) and 2-(3,5-dichlorophenyl)-5-(2,2,2-trifluoroethyl)oxazole-4-carboxylic acid (**111**) were found to possess the maximum activity. Results are mentioned in Table 44 [67].

**Table 44:** In vitro transthyretin binding selectivity assay

Compd.	Binding selectivity to transthyretin in human blood plasma
**110**	0.49 ± 0.07
**111**	0.68 ± 0.04

**Fig. 11:**
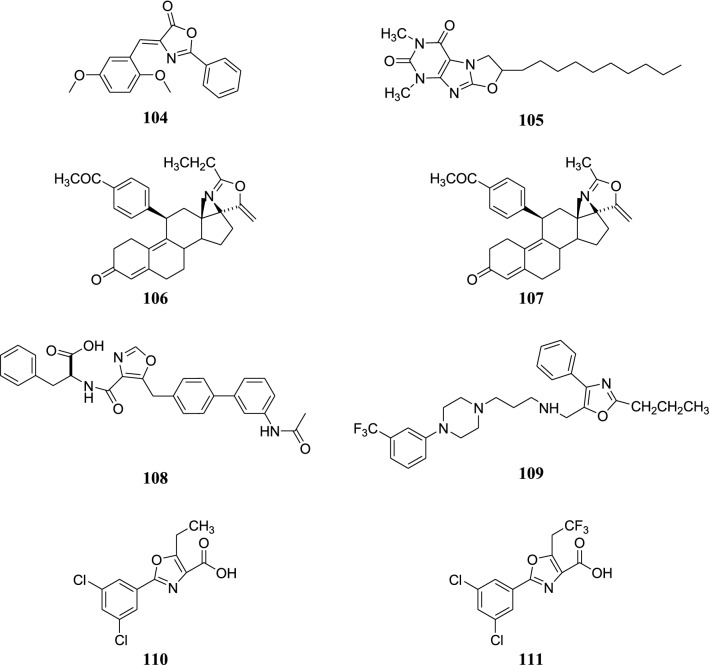
Structure of the most active antioxidant compound, adrenergic receptor ligand, antiprogesterone compounds, prostacyclin receptor antagonist, T-type calcium channel blocker and transthyretin (TTR) amyloid fibril inhibitors

The structures of the most active antioxidant compound (**104**), adrenergic receptor ligand (**105**), antiprogesterone compounds (**106**–**107**), prostacyclin receptor antagonist (**108**), T-type calcium channel blocker (**109**) and transthyretin (TTR) amyloid fibril inhibitors (**110**–**111**) are shown in Fig. 11.

## Conclusion

In summary, the present article aims to review the work reported on therapeutic potentials of oxazole derivatives which are valuable for medical applications during new millennium. This review article is based on synthesized oxazole derivatives which displays wide spectrum of biological potentials i.e. antibacterial, analgesic, anti-inflammatory, antidepressant, anticancer, antimicrobial, antidiabetic, antiobesity, antioxidant, adrenergic receptor ligand, antiprogesterone activity, prostacyclin receptor antagonist, T-type calcium channel blocker and transthyretin amyloid fibril inhibitory. The heterocyclic moiety being so versatile in nature offers the medicinal chemist to explore more about it in medicinal field and the data mentioned in this article will be a great help to prospective researchers working in this area for further study of this scaffold.

Oxazole moiety is an important heterocyclic compound as they are being an essential constituent of large number of marketed drugs. Having such diverse spectrum of biological activities, oxazoles has immense potential to be investigated for newer therapeutic possibilities and is an important class of lead compounds for development of new chemical entities (NCE) to treat various diseases of clinical importance.
